# Quantifying the Impact of Signal-to-background Ratios on Surgical Discrimination of Fluorescent Lesions

**DOI:** 10.1007/s11307-022-01736-y

**Published:** 2022-06-16

**Authors:** Samaneh Azargoshasb, Imke Boekestijn, Meta Roestenberg, Gijs H. KleinJan, Jos A. van der Hage, Henk G. van der Poel, Daphne D. D. Rietbergen, Matthias N. van Oosterom, Fijs W. B. van Leeuwen

**Affiliations:** 1 Interventional Molecular Imaging Laboratory, Department of Radiology, Leiden University Medical Center, Leiden, the Netherlands; 2 Department of Urology, Netherlands Cancer Institute-Antoni Van Leeuwenhoek Hospital, Amsterdam, the Netherlands; 3 Section of Nuclear Medicine, Department of Radiology, Leiden University Medical Center, Leiden, the Netherlands; 4 Department of Parasitology, Leiden University Medical Center, Leiden, the Netherlands; 5 Department of Infectious Diseases, Leiden University Medical Center, Leiden, the Netherlands; 6 Department of Urology, Leiden University Medical Center, Leiden, The Netherlands; 7 Department of Surgery, Leiden University Medical Center, Leiden, the Netherlands

**Keywords:** Image-guided surgery, Surgical robotics, Fluorescence imaging, Indocyanine green (ICG), Surgical performance

## Abstract

**Purpose:**

Surgical fluorescence guidance has gained popularity in various settings, e.g., minimally invasive robot-assisted laparoscopic surgery. In pursuit of novel receptor-targeted tracers, the field of fluorescence-guided surgery is currently moving toward increasingly lower signal intensities. This highlights the importance of understanding the impact of low fluorescence intensities on clinical decision making. This study uses kinematics to investigate the impact of signal-to-background ratios (SBR) on surgical performance.

**Methods:**

Using a custom grid exercise containing hidden fluorescent targets, a da Vinci Xi robot with Firefly fluorescence endoscope and ProGrasp and Maryland forceps instruments, we studied how the participants’ (N = 16) actions were influenced by the fluorescent SBR. To monitor the surgeon’s actions, the surgical instrument tip was tracked using a custom video-based tracking framework. The digitized instrument tracks were then subjected to multi-parametric kinematic analysis, allowing for the isolation of various metrics (e.g., velocity, jerkiness, tortuosity). These were incorporated in scores for dexterity (*Dx*), decision making (*DM*), overall performance (*PS*) and proficiency. All were related to the SBR values.

**Results:**

Multi-parametric analysis showed that task completion time, time spent in fluorescence-imaging mode and total pathlength are metrics that are directly related to the SBR. Below SBR 1.5, these values substantially increased, and handling errors became more frequent. The difference in *Dx* and *DM* between the targets that gave SBR < 1.50 and SBR > 1.50, indicates that the latter group generally yields a 2.5-fold higher *Dx* value and a threefold higher *DM* value. As these values provide the basis for the *PS* score, proficiency could only be achieved at SBR > 1.55.

**Conclusion:**

By tracking the surgical instruments we were able to, for the first time, quantitatively and objectively assess how the instrument positioning is impacted by fluorescent SBR. Our findings suggest that in ideal situations a minimum SBR of 1.5 is required to discriminate fluorescent lesions, a substantially lower value than the SBR 2 often reported in literature.

**Supplementary Information:**

The online version contains supplementary material available at 10.1007/s11307-022-01736-y.

## Introduction

In recent years, fluorescence-guided surgery has rapidly gained popularity. While many different fluorescent dyes have seen implementation in patients [1], surgical use of fluorescence is most often related to use of the near-infrared dye indocyanine green (ICG). This dye has been applied for diagnostic purposes in, for example, cardiology and ophthalmology since the late 1950s [2]. During the last decade surgeons have used ICG in applications such as lymph node (LN) mapping [3, 4], angiography (e.g., anastomosis) [5–8] and real-time identification of lesions (e.g., hepatobiliary lesions) [9, 10]. ICG is most extensively used during plastic surgery [11], urology [12] and gynecology [13, 14], but applications are rapidly expanding into general oncologic surgery [15], and head and neck surgery [16]. To facilitate ICG-based fluorescence guidance, established minimally invasive surgery platforms, such as laparoscopes and the da Vinci surgical robot, now include a near-infrared ICG imaging option [17, 18].

Generally, “free” or non-bound ICG is used for physiological imaging in doses of 5-25 mg/patient [19], thus providing relatively high local fluorescent concentrations. Fueled by the constant flow of nuclear medicine-based molecular imaging successes obtained with small molecules, peptides and antibodies, the field has moved toward the use of receptor-targeted fluorescent tracers. There are, however, some fundamental differences in the application of a receptor-targeted radiotracer at quantities below the micro-dosing level (< 100 µg/patient) [20] and use of a fluorescent-based receptor-targeted tracer at, e.g., 0.18 mg/kg [21]. For one nuclear medicine has a superior sensitivity and is capable of accurately detecting very low tracer quantities, hence the general compatibility with microdosing. The detection sensitivity for fluorescence is a.o. limited by light scattering and tissue attenuation. This means it not only is limited to superficial targets but also its sensitivity is inferior to that of nuclear medicine [21, 22]. Second, while the signal intensity at physiological imaging is directly related to the amount of tracer administered (in relation to the biological clearance half-life), the number of cell-surface receptors that can be targeted in a tumor volume is dictated by biology, at least in patients. Despite the positive effects that receptor internalization has on signal intensity, this still means there is a limit to the degree of tracer that can be accumulated in a tumor. One may even suggest that the accumulation of tracer in a tumor is fixed and can be calculated with, for example, positron emission tomography (PET) standard uptake volume (SUV) values. If that is the case it means that above certain doses the tracer uptake is no longer directly related to the quantity of tracer administered, while the increase in background signals in non-target tissues is. Combined this means that key challenges are the prevention of false negatives (e.g., lesions missed due to ‘underdosing’ or low camera sensitivity [22]) or false positives (e.g., due to mistaking the reflectance of excitation light as signal or background signals in non-target tissue as the result of ‘overdosing’ [23]). Knowing this, physics indicates that, unless the brightness of fluorescent dyes and the sensitivity of cameras improve radically, most receptor-targeted fluorescence-guided surgery applications will come with relatively low signal intensities and relatively high background signals. This will ultimately reflect on the signal-to-background ratio (SBR) and the ability of fluorescence imaging to guide surgical decision making.

It has been posed that image-guided surgery relies upon obtaining good SBRs [24]. As long-term outcome data and randomized clinical studies are still rare, often visualization of targets, with a SBR ≥ 2, is considered a surrogate endpoint for success [25, 26]. The significance of SBR values is further underlined by the rigorous pursuit of ways to increase the SBR. Approaches vary from using ‘therapeutic’ quantities of fluorescent tracers [21], to extended intervals between tracer administration and surgical imaging [27], and tuning of tracer pharmacokinetics to reduce local background [28]. More exotic approaches include the use of activatable dyes [29] or fluorescence lifetime imaging [29]. Despite all these efforts, as far as we know, there are no studies that describe how SBR values, and with that, fluorescence imaging, reflect on the surgical procedure itself. Meaning it is still unclear how a specific SBR value alters the way surgeons approach a target.

In robotic surgery most of the proficiency scoring is still qualitative (i.e., expert (video) assessment). Recent literature, however, suggests that objective and quantitative performance scoring can be realized by tracking mechanical movements of the robotic instruments (e.g., dVLogger Intuitive). A concept has even allowed first steps to be made to relate surgical performance to outcome [30–32]. Kinematic analysis has even become standard during virtual simulation training [33–35]. Recently, we reported that kinematics can also support the comparison between image guidance modalities [36]. We argue such quantitative assessments need to be inclusive for all laparoscopic and robotic surgery. With that in mind video-based isolation of kinematic metrics provides a universal and interchangeable solution that can easily be disseminated across different platforms.

Given the theoretical impact that fluorescence SBR has on surgical movement, we performed multi-dimensional kinematic analysis during a fluorescence guided surgery exercise. Following automated video assessments surgical instrument movements were digitized and analyzed in detail.

## Methods

### Phantom Design

To evaluate the sufficient fluorescent SBR needed for robotic surgery, we created a custom silicone (4 × 7) grid-phantom setup (Dragon Skin FXPro silicone, mixed with coloring pigment, FormX, Amsterdam, the Netherlands) that contained 28 possible target locations that could be sealed off with silicone lids. As targets we used 33.33 µL fluorescent beads [37], containing different concentrations of ICG (1–0.0625 mg/mL dissolved in methanol) incorporated in epoxy resin (ratio: 1:6:1 for epoxy resin, epoxy hardener and ICG solution, respectively).

#### Instrument Tracking Framework

**Fig. 1:**
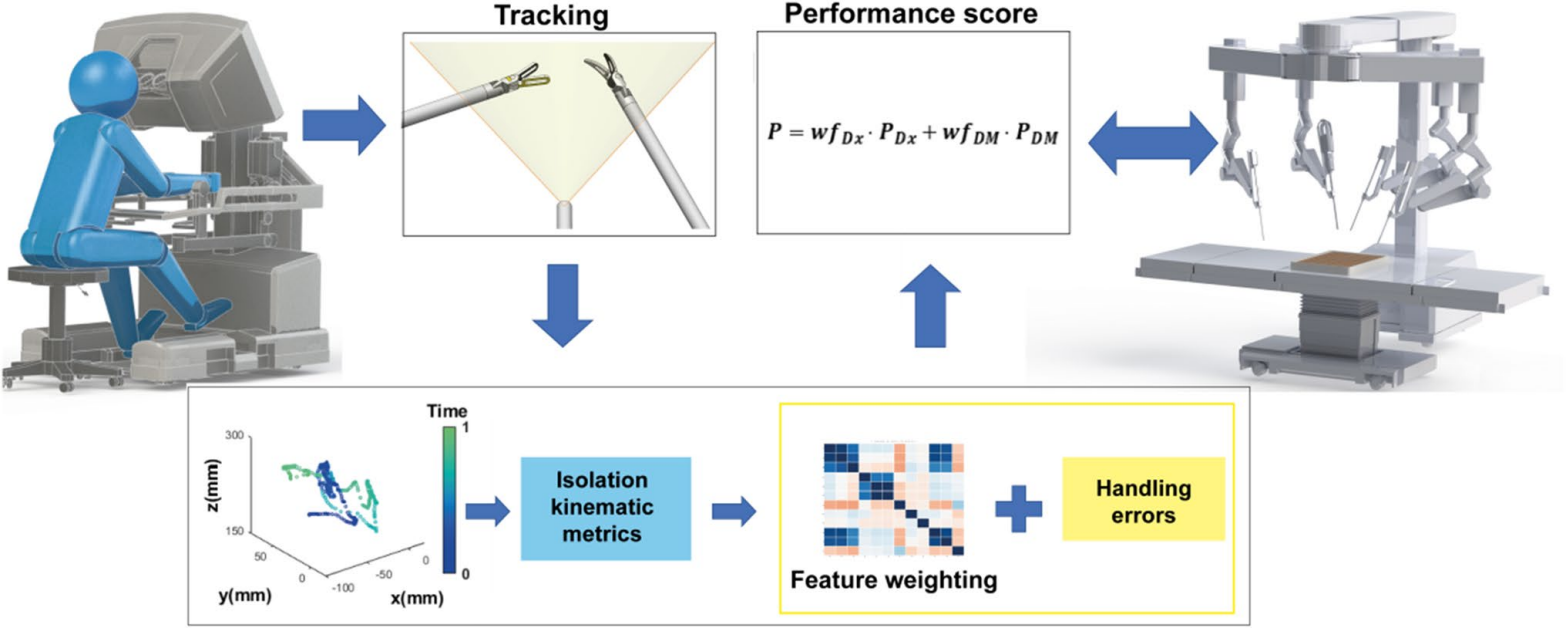
Schematic overview of the phantom-based study setup. Surgical instruments are tracked to be digitized for kinematic feature extraction. These features are then used to determine performance scores in relation to fluorescence signal-to-background ratios

**Fig. 2:**
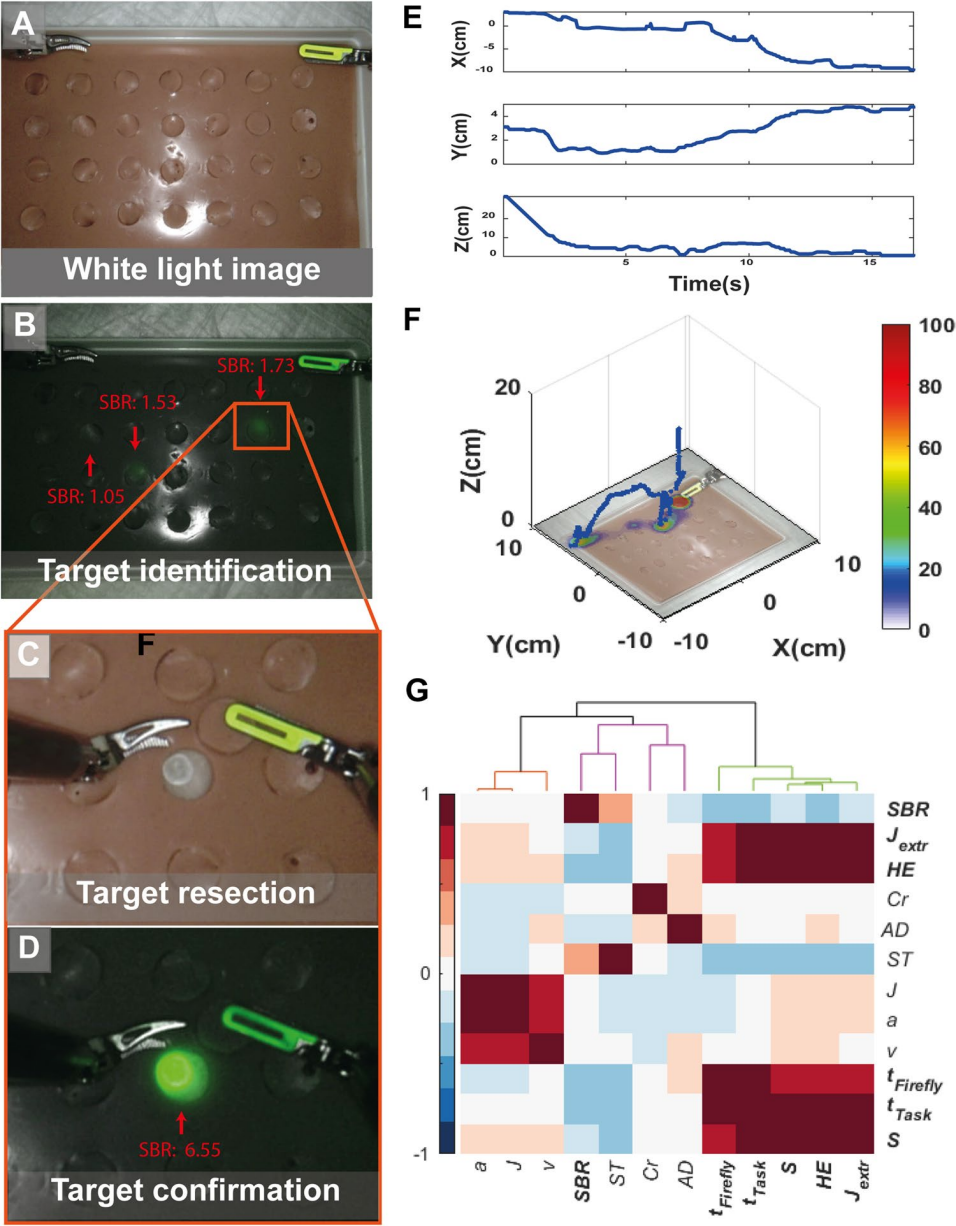
A-D) Experiment process consisting: A), start in white light image, B) target identification in Firefly fluorescence mode, C) target resection, D) confirm the right target location in Firefly fluorescence mode. E) X, Y, Z component of instrument movement resulting from customized tracking program, F) digitized traveled path of the ProGrasp instrument tip in 3D with position density (%s/cm^2^) plot on XY plane overlaid on phantom image and G) cluster analysis of Pearson correlation applied on the kinemetric metrics extracted from the traveled paths by the ProGrasp instrument tip, handling errors occurring during the exercises and SBR’s from all participants. The color bar implies the correlation strength between the features (red positive and green negative correlation).(SBR: signal-to-background ratio, J_ext_: number of extreme peeks in Jerkiness, HE: handling error, Cr: curvature, AD: angular dispersion, ST: straightness index, J = jerkiness, a = acceleration, v = speed, t_task_: task time, t_Firefly:_ Firefly fluorescent time, S: pathlength)

The surgical system used during these experiments was a da Vinci Xi robot with Firefly fluorescence chip-on-a-tip endoscope and ProGrasp™ and Maryland forceps instruments (Intuitive Surgical, Sunnyvale, CA; see Fig. 1). During the exercise as explained below, the ProGrasp served as the dominant instrument. To acquire the coordinate data of the ProGrasp™ instrument tip in 3-dimensional (3D) space, we made use of a previously described custom marker-based tracking framework [36]. In this setup the marker-based tracking accuracy proved to be 1.10 ± 0.74 mm, 0.50 ± 0.53 mm and 0.88 ± 0.99 mm in the x, y and z directions, respectively [36]. As marker a yellow-colored and rectangular-shaped marker was placed on one side of the ProGrasp (Fig. 2A). Matching computer-vision software was created to segment the markers (based on shape and color) in the endoscopic-video output. The obtained 3D instrument paths were then digitized using custom algorithms (constructed in MATLAB®, the MathWorks, Inc.). From the data we extracted multi-dimensional kinematic metrics such as spatial features (e.g., total pathlength, straightness index) and temporal features (e.g., time of completion, speed, acceleration, jerkiness). For better interpretation of the movement data, the percentage of time spent in each square centimeter of the Firefly image plane (%s/cm^2^) was calculated and used to calculate a color-coded density plot [36].

#### Fluorescence Intensity Determination

The SBR values of the individual targets in the grid phantom were determined using a color-based image segmentation algorithm created in MATLAB® on video material recorded from the Firefly camera at around 20 cm distance. In these algorithms, non-fluorescent but saturated parts (i.e., presented as white reflections) of the images were first removed using HSV (hue saturation value) color segmentation. Afterward, green fluorescent intensity was determined of each pixel in the image using the green color channel of the filtered RGB (red green blue) images. The target’s fluorescent intensity, and therefore signal value, is then determined as the mean pixel intensity value within a manual defined rectangle-shaped region of interest around the target’s location. For background calculation the average of three locations adjacent to the targets was used.

### Exercise

Participants (n = 16; MD’s (n = 4), researchers (n = 6), and engineers (n = 6) aged between 20 and 50) were asked to perform the exercise in which they had to locate and remove three randomly placed fluorescent targets from the grid phantom using the da Vinci Xi surgical system. Each participant was asked to perform one to five of such exercises, and the exercises did not end until all targets (three per exercise) were removed. This resulted in a total of three to fifteen fluorescent targets. The Firefly fluorescent imaging mode could be used to identify the target locations, but in line with clinical practice removal of the fluorescent beads had to take place in ‘white light’ mode. Non-visualization in fluorescent imaging mode meant the participant had to randomly check potential target locations in the 4 × 7 grid, thus substantially altering his/her movement kinematics. During the exercise, opening of locations not containing a fluorescent target, was considered a handling error. Exercises were timed, as was the use of fluorescence mode. To limit occlusions in instrument tracking, the participants were instructed to keep the instrument marker visible as much as possible during the exercises.

### Surgical Performance Scoring

Surgical performance is a sum of dexterity (*Dx*) and decision making (*DM)* [38]. In the exercise, the overall dexterity is represented by the normalized jerkiness (derivative of acceleration) in 3D over the entire procedural time from start (${t}_{1}$) to finish (${t}_{2}$) corrected for the pathlength (s). The *Dx* index can be calculated using Eq. 1 wherein a low dexterity index indicates a more focused performance [39].

1 $$Dx=\left({\int }_{{t}_{1}}^{{t}_{2}}{\left(\frac{{\delta }^{3}x}{\delta {t}^{3}}\right)}^{2}+{\left(\frac{{\delta }^{3}y}{\delta {t}^{3}}\right)}^{2}+{\left(\frac{{\delta }^{3}z}{\delta {t}^{3}}\right)}^{2}dt\cdot \frac{\Delta {t}^{5}}{{s}^{2}}\right)$$

The *DM* index can be described by a correlation between the intentional movements; sudden changes in dexterity (${\Delta Dx}_{extr}$), handling errors ($HE$) and procedural fluency ($F$), where a low $DM$ index indicates a more certain participant. The changes in dexterity are described by the number of extreme peaks within the jerkiness (#${J}_{extr}$; extreme peaks are above 100,000 m/s^3^). Procedural fluency $F$ is defined as a combination of the time spent in Firefly fluorescence mode (*t*_*Firefly*_) and straightness index (*ST*), as shown below.

2 $$DM={wf}_{1}\cdot {\Delta Dx}_{extr}+{wf}_{2}\cdot HE+{wf}_{3}\cdot F$$

$${\Delta Dx}_{extr}=\#{J}_{extr}$$

$$F={wf}_{3}\cdot {e}^{-\mathrm{log}\left(ST\right)}+{wf}_{4}\cdot {t}_{Firefly}$$

The weight factors (i.e., ${wf}_{1}$, ${wf}_{2}$, ${wf}_{3}$ and ${wf}_{4}$) in which each of the features contribute to the *DM* were determined using a maximization on the linear fit between the features and the total pathlength, based on the assumption that lesser movements resemble a more proficient procedure [40]. This was realized using a MATLAB® optimization program. Here the sum of the weight factors equals to 1; ${wf}_{1}+ {wf}_{2}+ {wf}_{3}+{wf}_{4}=1$ and $wf$ range between [0, 1] with step size 0.02.

Furthermore, an overall performance score (*PS*) can be created using the *Dx* and *DM* indices. This PS, ranging from 0 to 1, is depicted in Eq. 3, where a low score resembles a poor performance and is constructed in the following manner based on the method published by Ganni et al. [41]. To establish this *PS* range [0–1], the *Dx* and *DM* indices are linearly transformed into performance components ${P}_{Dx}$ and ${P}_{DM}$. This transformation was constructed in such manner that the median *Dx* value of the exercises with handling errors is resembled by a ${P}_{Dx}$ of 0.6 and the median *Dx* value of the exercises without handling errors is resembled by a ${P}_{Dx}$ of 0.9. The *DM* indices were transformed in an equal manner. The weightings in which the ${P}_{Dx}$ and ${P}_{DM}$ contribute to the *PS* were determined using a principal component analysis (PCA) wherein the sum of the weight factors equals to 1: ${wf}_{Dx}+ {wf}_{DM}=1$.

3 $$PS={wf}_{Dx}\cdot {P}_{Dx}+{wf}_{DM}\cdot {P}_{DM}$$

To determine what *PS* cutoff is considered to be proficient and therefore establish a proficiency level, the Z-score method can be used [42]. To avoid an underestimation of proficiency, only exercises showing no handling errors have been included in the Z-score calculation. Here the performance was rated proficient when the individual *PS* values are within a Z-score interval of [-2, 2] as is most common.

#### Statistics

Statistical significance between the performance was established via a two sampled t-test with the SPSS statistical software (IBM SPSS Statistics for Windows, Version 25.0), using a confidence interval of 95%.

## Results

### Kinematic Metrics Extraction of the Traveled Instrument Path and the Intra-feature Correlation

Figure 2A and B shows typical examples of white light and fluorescence imaging visualizations of the grid phantom, respectively. Figure 2C and D illustrates how opening of a lid can reveal a fluorescent target. During the exercises, SBRs of the targets as detected by the Firefly camera varied between 1.0027 and 3.4638 (total 120 targets). Analysis of the path traveled by the ProGrasp instrument helped to objectively evaluate the effect of SBR on the surgical proficiency (Fig. 2E and F). The position density in 2D (%s/cm^2^) could be visualized through color coding. To get an indication in which location instrument time was largest, this value could be overlaid onto the phantom, see Fig. 2F x-, y- plane. Following the direct relation between SBR values and kinematic metrics extracted from the digitized instrument trajectories (Pearson correlation coefficient; Fig. 2G), we observed that the SBR is negatively correlated with pathlength, time to complete the task, time spent in Firefly fluorescent mode and number of handling errors. Positive correlations, in which the two variables move in tandem, are found for SBR and straightness index. Also, pathlength, task time, time spent in Firefly fluorescent mode are positively correlated with the number of handling errors and extremes in jerkiness.

**Fig. 3:**
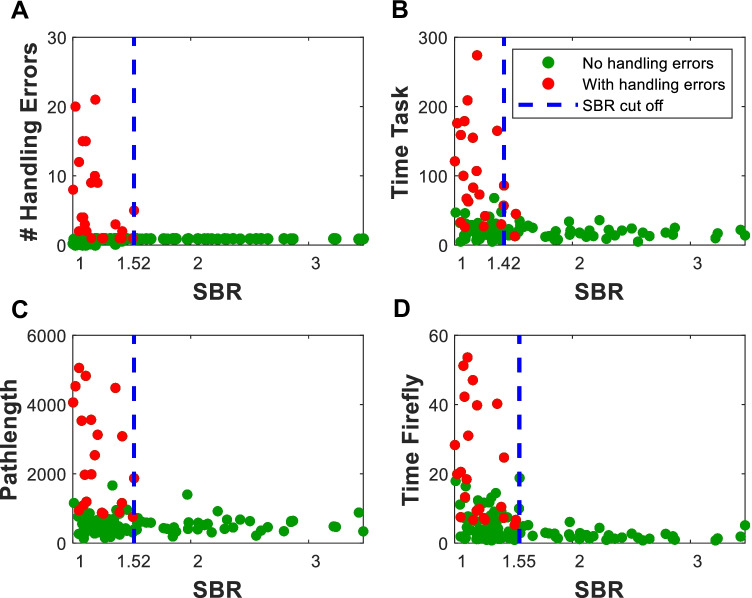
A) The relation between SBR and handling errors and the sufficient SBR of 1.52. A similar setup for the relation between the SBR and procedural time (B), total pathlength (C) and the time spend in Firefly fluorescence mode (D) with sufficient SBR of 1.42, 1.52 and 1.55, respectively

In general, minimizing handling errors is considered a critical aspect of refining surgical procedures [43, 44]. Hence, we grouped the exercises into sub-classes with and without handling errors. Relating these handling errors to the SBR values, helped identify what SBR support selective target removal and how it affects dexterity and decision making. Figure 3 shows that handling errors, total procedural time, total pathlength, as well as time spent in Firefly fluorescence mode were SBR dependent. The cutoff SBRs were defined where sudden change is observed in these features and results in SBR = 1.52, SBR 1.42, SBR 1.52 and SBR 1.55, respectively. Averaging these values indicates that the ‘sweet spot’ for target identification means SBR > 1.50 is desirable. At lower SBR values participants increased the number of handling errors which also converted to increased pathlength ${(S}_{avg, SBR<1.50}=1256$ vs ${S}_{avg,SBR>1.50}=568.3 \mathrm{mm})$ and task time (${t}_{Tas{k}_{avg}, SBR<1.50}=99.57$ vs ${t}_{Tas{k}_{avg}, SBR>1.50}=19.77 \mathrm{s}$). The uncertainty of the target location also meant participants spent more ‘search’ time in Firefly fluorescence mode ${(t}_{Firefl{y}_{avg},SBR<1.50}=10.23$ vs ${t}_{Firefl{y}_{avg},SBR>1.50}=3.12 \mathrm{s}$). In some cases, however, the increased time in fluorescence mode did mean the targets could still be identified (see Fig. 3D).

### The Relation Between SBR and Surgical Performance Scoring

**Fig. 4:**
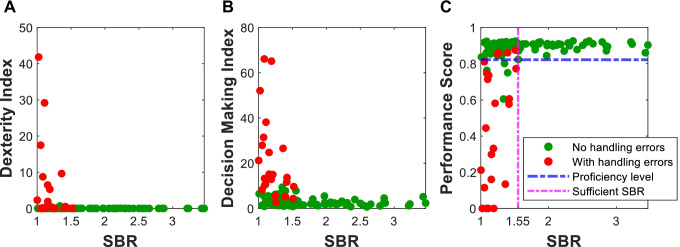
A)The dexterity index, B) decision-making index and C) the overall performance score plotted against the SBR. This clearly shows with SBR > 1.55 the performance score is mostly above proficiency level with no handling errors (green)

From the kinematic metrics and handling errors we were able to determine the $Dx$ and $DM$ indices, which are positively correlated with the total pathlength. Note: Pathlength and completion time are the most common measure for performance indicated in the literature [45–47]. Figure 4A and B suggests that lowering of the SBR converts to a decreased performance as indicated by a higher $Dx$ and $DM$. A comparison between SBR < 1.50 and SBR > 1.50 groups suggests that the latter group generally yields a 2.5-fold higher $Dx$ value and a threefold higher $DM$ value (${Dx}_{avg,SBR>1.50}=3.75$ vs ${Dx}_{avg,SBR<1.50}=9.20$ and ${DM}_{avg,SBR>1.50}=2.34$ vs ${DM}_{avg,SBR<1.50}=8.09$). To score the performance (*PS*), these indices were weighted according to the PCA results, yielding ${wf}_{Dx}=0.49$ and ${wf}_{DM}=0.51$ (see Fig. 4C). Participants were considered proficient at $PS$ ≥ 0.816. Relating proficiency to the SBR, shows a SBR cutoff at 1.55 (Fig. 4C, pink line) above which all participants performed proficient. Note fully, some individuals still performed proficient even when showing handling errors. However, none of the participants showing multiple handling errors reached the proficiency level. There is a difference between the average *PS* of the exercises showing no handling errors (Fig. 4C; green) and the exercises with handling errors (Fig. 4C; red) (${PS}_{avg}=0.888$ vs. ${PS}_{avg}=0.509;p =0.06$). Furthermore, no handling errors occurred above a SBR of 1.53.

In the current study it was not possible to relate the backgrounds of the individuals to the performance, MD’s (${PS}_{median}=0.7920$), researchers (${PS}_{median}= 0.8245$) and engineers (${PS}_{median}= 0.8641$; $p =0.27$). If anything, the trend suggests the technical expertise/insight of the participant reflects positively on the score.

## Discussion

By analyzing the kinematics of surgical instrument movement, it has become possible to objectively and quantitatively define below which SBR the value of fluorescence guidance starts to deteriorate. Our findings indicate that the cutoff lies around SBR > 1.5 (average value calculated from cutoffs indicated in Fig. 3 and 4). This value is markedly lower than the artificial SBR cutoff value of 2 that is often used in the literature [48, 49]. We argue that studies such as the one presented here help gain fundamental insight in the fluorescence signal intensities that are needed to guide surgeons in their actions. 

We observed that lower concentration of fluorescent contrast showed impact on a variety of kinematic metrics, namely, time in Firefly fluorescence mode, time of task completion and total pathlength of the movement of the participant. Not only do these metrics relate to each other, feature correlation analysis also indicates they relate to: SBR, handling errors, extremes in jerkiness and straightness index (Fig. 2G). Below the SBR 1.5 cutoff value the decision-making process is negatively impacted by the operators inability to discriminate between the reflection of excitation light and low-intensity fluorescent emissions. Interestingly, this means that at low signal intensities also more non-targets are pursued (so-called false positives) and handling errors become more frequent. In actual surgery, false positives can of course also be caused by accumulation of a fluorescent tracer in non-target tissue.

Surgical training literature relies heavily on handling errors for performance scoring [50]. Our findings indicate that participants could show a high proficiency ($PS \ge 0.816$), despite having a single handling error (Fig. 4C). Since lower fluorescence intensities in targets automatically convert to a drop in SBR, there will be a direct link between our phantom data and clinical findings that indicate that the accuracy of fluorescence guidance deteriorates with decreasing fluorescence concentrations (for ICG in SN surgery, the concentration threshold has been defined as < 0.003 nmol/cm^3^) [21]. It should however be noted that the exercises that we used present a quite ideal situation viz. no bleeding. Therefore *in vivo* SBR values could be slightly different. Hence, implementing the SBR analysis and kinematic scoring in clinical trials is required to validate if the current SBR threshold directly translates to the *in vivo* situation. This is something that is currently under investigation. Hereby we should realize that the kinematic scoring of instrument movements only relates to the SBR values encountered in situ. Values that in general are much lower than values determined during back table analysis. For example, in their study Moore et al. indicated in situ fluorescent SBR values in the range 1.5–2.5 converted to much higher SBR values *ex vivo* (range 3.0–4.0) [51]. Recently, de Barros et al. reported a similar trend for radioguidance [52].

We based our current study on the commonly used fluorescent dye ICG and the da Vinci platform equipped with a Firefly fluorescence camera that is optimized to identify this particular dye. As such our findings reflect on a situation wherein all parameters are fully optimized. As mentioned before in the clinic multiple fluorescent dyes have been used [1], and there have been reports on using the Firefly camera for other dyes such as fluorescein [53] and IRDye800CW [54]. In these situations, the fluorescent emission may not be optimally matched to the camera. As such the SBR values may be structurally lower. However, assuming the fluorescence is displayed to the surgeon in the same (artificial) green coloration with black and white background, the interpretation of the images and this SBR values will remain identical. That said, representation of the fluorescent emission in another coloration, e.g., white [55] or blue [56] or heatmap [48, 57], may change the interpretation of the surgical field, as will the display of white light images in conjunction with fluorescence [58].

Almost half the study participants could identify targets and show a proficient performance below the SBR 1.5 cutoff (Fig. 4C), but their accuracy dropped from 100% to 78.95%. This underscores that individual vision can play an important role in overall performance. A difference that did not relate to the background of the individuals. It still needs to be defined if training in fluorescence imaging could help enhance a surgeon’s ability to identify low-intensity fluorescent hotspots. This requirement may, however, become obsolete in the future when neural networks take over such tasks and automatically analyze fluorescence signal intensities at a per pixel-level basis and a video-rate speed [59–61]. A similar effect has been reported for the surgeon’s ability to perceive objects in 3D [62–65]. For the latter training and selection programs have already been proposed [62].

Our findings show some limitations. For one, we performed our initial evaluations in a phantom setup, rather than in clinical trials. The reason for this is the ease of instrument tracking [36, 66] and the ability to have controlled SBR values. Here it should be noted that the setup used (epoxy fluorescent beads in a silicon phantom) does not allow us to translate the SBR values detected by the camera to fluorescence concentrations in the targets or the attenuating effect of ‘tissues’ covering a target, for that we refer to previous studies [21, 53]. Furthermore, in this study, the efficiency of the task is based on the movements of the dominant hand only. Future studies should determine whether the same trends extend to both hands. Obviously, handling errors in phantom exercises do not per se translate to a potential surgical complication and thus may appear somewhat artificial. During actual surgery on patients, however, resecting healthy tissue may lead to complications. In these instances, the anatomical location of the target will strongly influence the impact that handling errors yield. Hence the number of potential handling errors should be expanded with a score of severity, meaning the *DM* and *PS* scores should be tailored specifically to these applications.

## Conclusion

Through multi-parametric kinematic analysis and performance scoring we have gained practical insight in how fluorescence SBRs impact on the ability to surgically resect a target, indicating that a SBR > 1.5 is required. Given that the quest for receptor-targeted fluorescent agents approaches the use of signal intensities at the boundaries of what is technically feasible, such insights may help refine the implementation of fluorescence guidance in clinical trials and during routine care. Ultimately, they may also help guide the further development of fluorescent tracers and fluorescence imaging modalities.

## Supplementary Information

Below is the link to the electronic supplementary material.

Supplementary file1 (MP4 64969 KB)
